# Sex-Specific Urinary Biomarkers for Diagnosing Bipolar Disorder

**DOI:** 10.1371/journal.pone.0115221

**Published:** 2014-12-22

**Authors:** Jian-jun Chen, Hua Huang, Li-bo Zhao, De-zhi Zhou, Yong-tao Yang, Peng Zheng, De-yu Yang, Peng He, Jing-jing Zhou, Liang Fang, Peng Xie

**Affiliations:** 1 Department of Neurology, Yongchuan Hospital of Chongqing Medical University, Chongqing, China; 2 Department of Neurology, The First Affiliated Hospital of Chongqing Medical University, Chongqing, China; 3 Chongqing Key Laboratory of Neurobiology, Chongqing, China; 4 Institute of Neuroscience, Chongqing Medical University, Chongqing, China; Rutgers University, United States of America

## Abstract

Sex-based differences are prominent in affective disorders, but there are no biomarkers available to support sex-specific, laboratory-based diagnostics for male and female bipolar disorder (BD) patients. Here, a NMR-based metabonomic approach was used to preliminarily identify sex-specific urinary metabolite biomarkers for diagnosing male and female BD patients. A male-specific biomarker panel consisting of four metabolites (α-hydroxybutyrate, choline, formate, and N-methylnicotinamide) effectively discriminated between male BD and healthy controls (HC) subjects, achieving an area under the receiver operating characteristic curve (AUC) of 0.942. A female-specific biomarkers panel consisting of four metabolites (α-hydroxybutyrate, oxalacetate, acetone, and N-methylnicotinamide) effectively discriminated between female BD and HC subjects, achieving an AUC of 0.909. The male-specific biomarker panel displayed low discriminatory power in the female group, and the female-specific biomarker panel displayed low discriminatory power in the male group. Moreover, several other metabolites showed different trends between male and female BD subjects. These findings suggest that male and female BD patients have distinct biomarker fingerprints and that these two sex-specific biomarker panels may serve as effective diagnostic tools in distinguishing male and female BD patients from their healthy counterparts. Our work may provide a window into the mechanisms underlying the pathoetiology of BD in both men and women.

## Introduction

Bipolar disorder (BD), also known as bipolar affective disorder, manic-depressive disorder, or manic depression, is a debilitating mental disorder that affects up to 1% of the general population [1] and is typically characterized by episodes of elevated mood known as mania alternating with episodes of depression. Manic and depressive episodes can impair the individual's ability to function in ordinary life. The causes of BD vary between individuals, and the exact mechanism underlying the disorder remains unclear [2]. Currently, there is no objective laboratory-based testing for BD, so diagnosis relies solely on the subjective identification of clinical symptoms, which often results in delayed diagnosis or misdiagnosis. This phenomenon may contribute to increased suicide risk and poorer prognosis for BD patients [3], [4].

Metabonomics, which identifies and quantifies metabolites in various biofluids such as plasma/serum and urine, has been widely applied to define the metabolic perturbations in various disease states and thus facilitates identification of novel disease-specific biomarkers [5]. Metabonomics has been successfully used in identifying novel biomarkers for neuropsychiatric disorders, such as schizophrenia and autism [6], [7]. Nuclear magnetic resonance spectroscopy (NMR), one of three major analytical platforms with proven utility in metabonomic applications, has proven to be a well-established strategy for identifying disease-specific biomarkers because of its highly sensitive, high-throughput molecular screening capability [8]. Using this method, our group has identified a panel of potential metabolite biomarkers capable of discriminating major depressive disorder (MDD) subjects from healthy controls (HC) with both high sensitivity and specificity [9], [10].

In regards to BD, several researchers also performed studies using NMR-based metabolomic platforms. Lan et al. found that an imbalance of excitatory/inhibitory neurotransmission may contribute to the etiopathogenesis of BD [11]. Sussulini et al. found a plasma metabolite signature was capable of discriminating BD patients from HC [12]. Using a NMR-based metabolomic platform, our group has identified a panel of metabolite biomarkers capable of discriminating BD subjects from HC with a sensitivity of 79.1% and a specificity of 79.2% [13].

However, these previous studies have not taken sex-based differences into consideration. Sex-based differences are prominent in affective disorders and may provide a window into the mechanisms underlying the pathoetiology of affective disturbances in both sexes. Although BD has similar prevalences in both sexes, men and women have different disease presentations and courses. First, females and male BD patients have different ages of onset [14]. Second, lobular volumes were not different in BD patients relative to HC, but there were unpredicted significant sex-by-diagnosis interactions in the left frontal and temporal and right parietal and occipital lobes, such that, relative to controls, male patients had larger volumes than females [15]. Third, when using the biomarkers identified in our previous study [13] to discriminate male BD patients from male HC and female BD patients from female HC, the results were significantly different. The potential biomarkers could effectively discriminate male BD patients from male HC with high sensitivity (83.3%) and specificity (90.6%), but not female BD patients from female HC. The sensitivity and specificity of the latter was only 77.3% and 60.5%, respectively.

Based on the aforementioned findings, it would be advisable to take sex-based differences into consideration when attempting to identify potential biomarkers for diagnosing BD. Therefore, in this study, a NMR-based metabonomic approach was applied to preliminarily identify sex-specific urinary metabolite biomarkers for separately diagnosing male and female BD patients.

## Materials and Methods

### Ethics Statement

Prior to sample collection, written informed consents were obtained from all recruited subjects. The protocols of this study were reviewed and approved by the Ethical Committee of Chongqing Medical University.

### Clinical Sample Selection

Forty-two male and forty-four female BD subjects presenting with manic, euthymic, or depressed states were recruited in the psychiatric center of the First Affiliated Hospital at Chongqing Medical University (Chongqing, China). All diagnoses were based on the Structured Clinical Interview from the DSM-IV-TR. About 72% of BD subjects were currently undergoing treatment. Briefly, they were treated with floxetine, paroxetine, citalopram, risperidone, olanzapine, and lithium. Subjects were excluded on the grounds of any pre-existing physical or other mental disorders. Subjects were also excluded for illicit drug use.

During the same time period, fifty three male and forty three female HC subjects were recruited from the medical examination center of the First Affiliated Hospital at Chongqing Medical University. Exclusion criteria for HC candidates included any current or previous lifetime history of neurological, DSM-IV Axis I/II, and/or systemic medical illness. Each subject gave oral and written informed consents after being fully informed about the study.

### Urinary Sample Preparation

Morning urine samples after overnight fasting were collected in a sterile cup and transferred into a sterile tube. The resulting supernatant, after centrifugation at 1500 g for 10 min, was immediately divided into equal aliquots and stored at −80°C.

### NMR Acquisition

Prior to NMR analysis, urine samples were thawed and centrifuged at 1500 g for 10 min to remove precipitation. To ensure stabilization of urinary pH, 500 µl of urine was mixed with 100 µl of phosphate buffer (90% D2O, 1 mM 3-trimethylsilyl-1-[2,2,3,3-^2^H4] propionate (TSP), and 3 mM sodium azide; pH 7.4). After centrifugation at 12000 rpm for 10 min, 500 µl samples of supernatant were transferred into 5 mm NMR tubes. The proton spectra of the urine samples were collected on a Bruker Avance 600 spectrometer operating at a 600.13 MHz 1H frequency with a standard 1-dimensional (1D) pulse sequence. Typically, 64 transients were collected into 16K data points with a spectral width of 8000 Hz, an acquisition time of 0.945 s, and a relaxation delay of 2 s. Prior to Fourier transformation, the free induction decay (FID) was zero-filled and multiplied by an exponential function corresponding to a line-broadening factor of 0.3 Hz in frequency domain. Urine resonance assignments were performed according to the previous literature and NMR databases [6], [10].

### NMR Spectral Processing

We used the 3-trimethylsilyl-1-[2,2,3,3-^2^H4] propionate (TSP) as an internal standard to confirm the change in the urinary levels of the metabolites. All spectra were manually phased and baseline referenced to TSP resonance at δ0.0. The NMR spectra (0.5–9.5 ppm) were segmented into equal widths (0.005 ppm) using the AMIX package (Bruker Biospin, Germany). Spectral regions of water and urea resonances were excluded before analysis to eliminate baseline effects of imperfect water saturation.

### Key Metabolite Selection

The integral values of samples were imported into SIMCA-P+ 12.0 software (Umetrics, Umeå, Sweden) as variables for multivariable statistical analysis. Orthogonal partial least squares discriminant analysis (OPLS-DA) was applied to the unit variance (UV)-scaled spectral data to visualize discrimination between BD subjects and healthy controls [16]. Compared to Pareto scaling, UV scaling possessed a higher weight of lower concentration metabolites. The coefficient loading plots and variable importance (VIP) of the OPLS-DA model were used to identify the spectral variables contributing to sample discrimination on the scores plot [17], [18]. The metabolites with VIP values of no less than 1 and a correlation coefficient of |r|>0.246 (equivalent to a p-value of less than 0.1) were identified as key metabolites responsible for sample differentiation and then entered into a multivariate logistic-regression model. Furthermore, a 199-iteration permutation test was performed to rule out non-randomness of separation between groups.

### Metabolite Biomarker Identification

As clinical diagnosis based on the quantification of a small number of metabolites would be more practical, a forward step-wise binary logistic regression algorithm based on Bayesian Information Criterion (BIC) was used to optimize the metabolite biomarker combination. This algorithm can identify a minimum number of potential biomarkers and construct a discriminative model using these biomarkers. Using this model, the probability of illness in each sample can be calculated. Then, a receiver-operating characteristic (ROC) curve and area under the receiver operating characteristic curve (AUC) values can be obtained from the disease probability values [19]. SPSS 19.0 software was used to perform these analyses.

## Results

A total of eighty-six BD subjects and ninety-six HC subjects were recruited into this work. The detailed information was described in Table 1. There were no significant differences in the demographic variables of age and body mass index (BMI) between male BD and HC subjects as well as female BD and HC subjects. There were no significant differences in the number of BD subjects on medication between the male and female BD patient group. The detailed original data was in S1 File in the supporting information section.

**Table 1:** Demographic and Clinical Characteristics of BD Subjects and Controls^a^.

	Male set			Female set		
	HC	BD	*P*-value^b^	HC	BD	*P*-value^b^
**Sample Size**	53	42	–	43	44	–
**Age (years)** c	28.4±7.9	27.7±10.6	0.72	32.3±10.4	30.4±11.8	0.43
**BMI** c	21.4±2.7	21.9±2.3	0.32	21.8±2.6	21.5±2.5	0.61
**Depressed BD**	–	31	–	–	33	–
**Euthymic BD**	–	7	–	–	10	–
**Manic BD**	–	4	–	–	1	–

a Abbreviations: HC: healthy controls; BD: bipolar disorder patients; BMI: Body Mass Index

b Two-tailed student t-test for continuous variables (age and BMI)

c Values expressed as means ± SDs.

### Univariate Analysis

The non-parametric Mann-Whitney U test was used to detect statistical differences between BD and HC groups. A p-value of less than 0.05 was considered to be statistically significant. The false discovery rate was controlled according to the Bonferroni step-down procedure [20]. Eight metabolites had a higher level in female BD subjects but a lower level in male BD subjects. The detailed information is described in Table 2. Acetate had a lower level in female BD subjects but a higher level in male BD subjects. Oxalacetate had a significantly higher level in female BD subjects, but a non-significantly higher level in male subjects. Taurine had a significantly higher level in male BD subjects but a non-significantly higher level in female subjects.

**Table 2:** Distinct Metabolites for Males and Females with Bipolar Disorder.

		N	Iso	Hip	For	Cit	Ala	Atn	DHM	Ace	Oxa	Tau
**M**	*P*-value^a^	0.336	0.027	0.671	0.206	0.284	0.416	0.667	0.709	0.175	0.113	0.031
	FC^b^	0.486	0.516	0.040	0.405	0.289	0.244	0.419	0.043	−0.258	−0.376	−0.352
**F**	*P*-value^a^	0.491	0.983	0.005	0.737	0.265	0.614	0.079	0.090	0.055	0.033	0.545
	FC^b^	−0.347	−0.063	−0.809	−0.011	−0.445	−0.139	−0.712	−0.592	0.031	−0.688	−0.389

M: male; F: female; N: nicotinate; Iso: isobutyrate; Hip: hippurate; For: formate; Cit: citrate; Ala: alanine; Atn: acetone; DHM: 3, 4-dihydroxymandelate; Ace: acetate; Oxa: oxalacetate; and Tau: taurine.

a *P*-values were derived from two-tailed Student's *t*-test.

b Fold-change (log base 2). Positive values indicate lower levels in BD subjects, and negative values indicate higher levels in BD subjects.

### Key Urinary Metabolites

**Figure 1:**
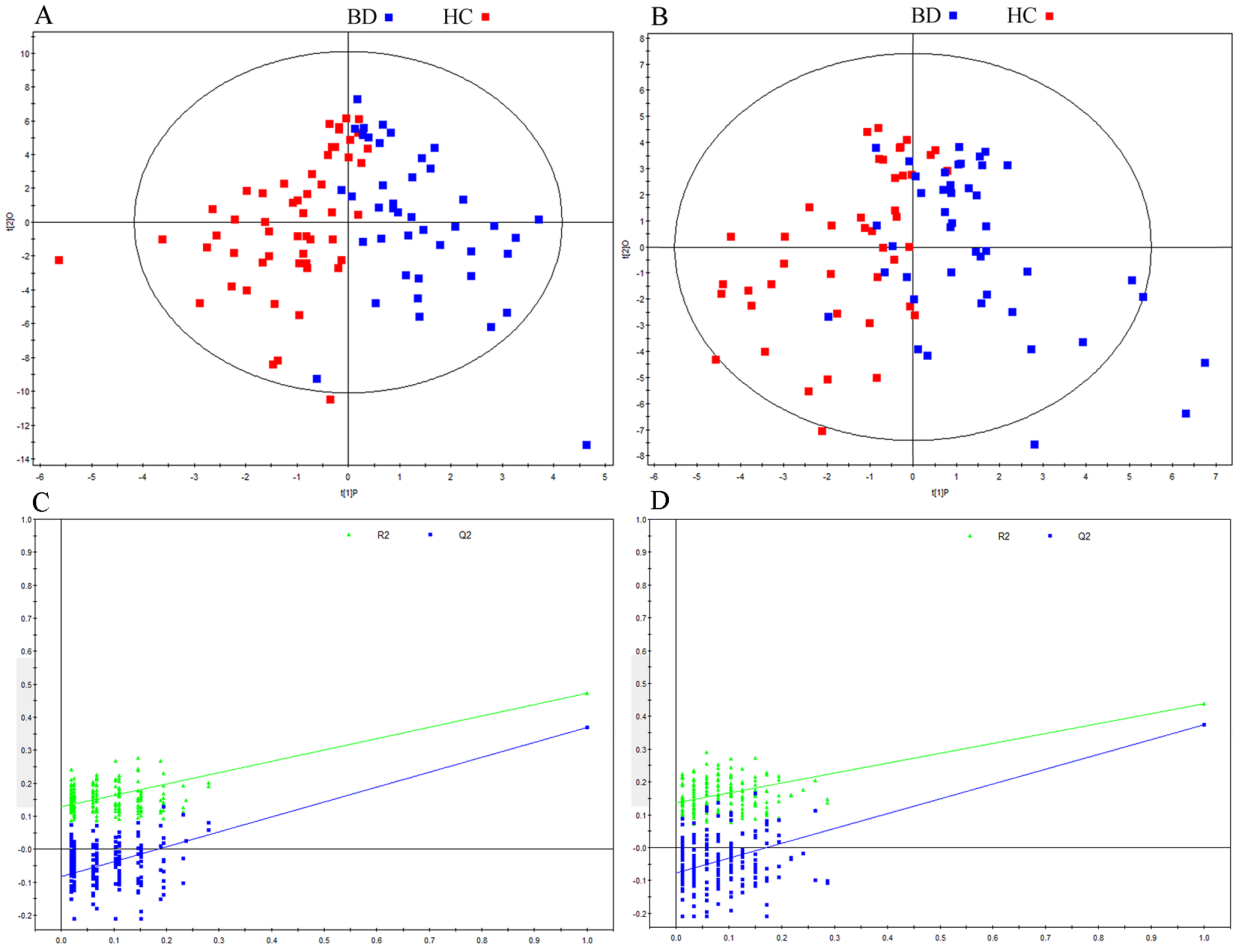
Metabonomic Analysis of Urine Samples. Orthogonal partial least-squares discriminant analysis (OPLS-DA) score plots showing a clear discrimination between (A) male BD subjects (red box) and male HC (blue box); (B) female BD subjects (red box) and female HC (blue box); (C) 199- iteration permutation test for male OPLS-DA model; (D) 199- iteration permutation test for female OPLS-DA model.

The resulting scores plot of the OPLS-DA model shows a clear discrimination between male BD and HC subjects (Fig. 1A) as well as female BD and HC subjects (Fig. 1B). The detailed information was described in Table 3. For males, the coefficient loading plots of the OPLS-DA model identified 13 differential metabolites, and the corresponding OPLS-DA loading plots identified nine differential metabolites. We finally identified nine differential metabolites responsible for distinguishing male BD subjects from HC. For females, the coefficient loading plots of the OPLS-DA model identified 18 differential metabolites, and the corresponding OPLS-DA loading plots identified 11 differential metabolites. We finally identified 11 differential metabolites responsible for distinguishing female BD subjects from HC. Furthermore, the permutation test showed the two constructed OPLS-DA models were valid and not-over fitted (Fig. 1C, D).

**Table 3:** Key Metabolites Responsible for Discriminating BD Subjects from HC.

No.	Metabolite	Sex	*P*-value^a^	VIP^b^	r^c^	Fold change^d^
**1**	Choline	Male	3.99×10^−3^	1.63	−0.52	−0.57
**2**	Dimethylglycine	Male	7.43×10^−2^	1.04	−0.31	−0.39
**3**	Formate	Male	2.06×10^−1^	1.01	0.54	0.41
**4**	Glyceroylphosphocholine	Male	1.02×10^−3^	1.94	−0.61	−0.98
**5**	Isobutyrate	Male	2.76×10^−2^	1.13	0.51	0.52
**6**	N-Methylnicotinamide	Male/Female	1.73×10^6^/8.97×10^−3^	2.11/1.69	−0.76/−0.73	−1.74/−1.34
**7**	Dimethylamine	Male/Female	2.25×10^−2^/1.19×10^−2^	1.32/1.43	−0.42/−0.37	−0.46/−0.85
**8**	m-Hydroxyphenylacetate	Male/Female	6.43×10^−3^/4.33×10^−3^	1.43/1.47	−0.41/−0.45	−0.52/−0.87
**9**	α-Hydroxybutyrate	Male/Female	2.28×10^−1^/7.42×10^−1^	1.82/1.00	0.27/0.83	1.49/1.07
**10**	Acetone	Female	7.98×10^−2^	1.69	−0.73	−0.71
**11**	Hippurate	Female	5.17×10^−3^	1.17	−0.44	−0.81
**12**	Malonate	Female	4.59×10^−3^	1.58	−0.52	−0.88
**13**	Oxalacetate	Female	3.35×10^−2^	1.20	−0.25	−0.69
**14**	Phenylacetyglycine	Female	3.97×10^−3^	1.42	−0.44	−0.83
**15**	p-Hydroxyphenylacetate	Female	9.08×10^−3^	1.39	−0.38	−0.69
**16**	Trimethylamine-N-oxide	Female	4.39×10^−3^	1.55	−0.46	−0.92

a *P*-values were derived from non-parametric Mann-Whitney U test.

b Variable importance in the projection (VIP) was obtained from OPLS-DA with a threshold of 1.0.

c Correlation coefficient was obtained from OPLS-DA with a threshold of 0.246. Positive values indicate lower levels in BD subjects, and negative values indicate higher levels in BD subjects.

d Fold change (log base 2). Positive values indicate lower levels in BD subjects, and negative values indicate higher levels in BD subjects.

### Urinary Metabolite Biomarkers

In order to obtain a simple urinary metabolite biomarker panel that would be useful in diagnosing BD in clinical practice, all key differential metabolites contributing to the discrimination between BD subjects and HC were considered as candidates and used to perform stepwise binary logistic regression analysis. A step-wise optimization algorithm based on Bayesian Information Criterion (BIC) was used to determine the minimum number of urinary metabolite biomarkers for BD.

**Figure 2:**
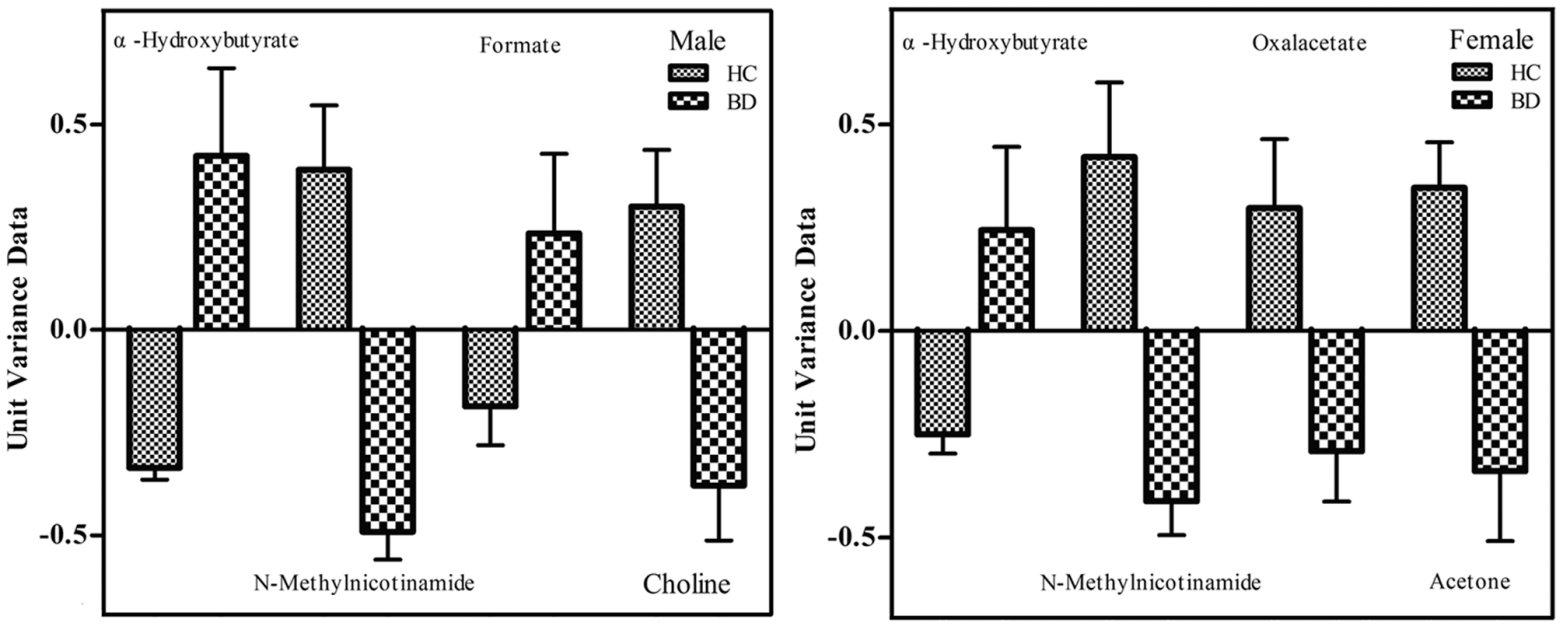
Key Metabolites Contributing to the Most Significant Deviations Between BD and HC Subjects.

**Figure 3:**
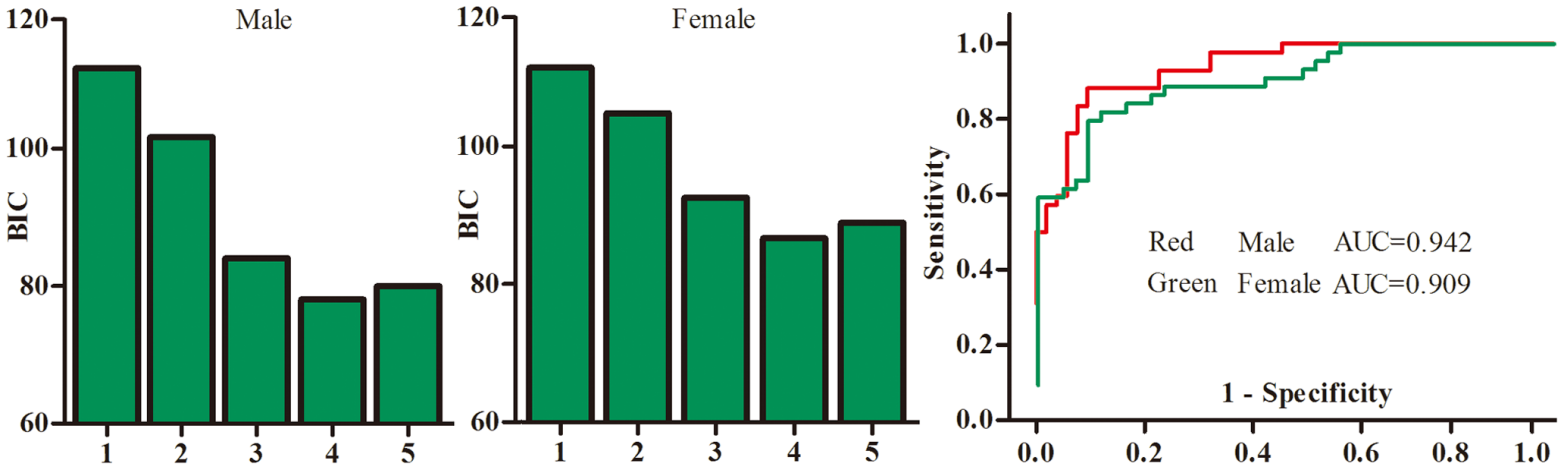
Identification and Validation of a Sex-Specific Urinary Metabolite Panel.

For males, four metabolites contributed to the most significant deviations between male BD subjects and HC, suggesting that these four metabolites could yield the highest predictive power for future diagnostic applications. These four metabolites were N-methylnicotinamide, formate, α-hydroxybutyrate, and choline (Fig. 2). Next, ROC analysis of a urinary metabolite biomarker panel composed of the foregoing four metabolites was performed to examine its utility in the diagnosis of BD. This biomarker panel was capable of discriminating 42 male BD subjects from 53 male HC with an area under the curve (AUC) of 0.942 (Fig. 3). The sensitivity and specificity were 85.7% and 90.6%, which was comparable to the sensitivity (83.3%) and specificity (90.6%) obtained by the original biomarker panel in our previous study [13].

For females, four metabolites contributed to the most significant deviations between male BD subjects and HC, suggesting that these four metabolites could yield the highest predictive power for future diagnostic applications. These four metabolites were N-methylnicotinamide, acetone, α-hydroxybutyrate, and oxalacetate (Fig. 2). Next, ROC analysis of a urinary metabolite biomarker panel composed of the foregoing four metabolites was performed to examine its utility in the diagnosis of BD. This biomarker panel was capable of discriminating 44 male BD subjects from 43 male HC with an area under the curve (AUC) of 0.909 (Fig. 3). The sensitivity and specificity were 81.8% and 83.7%, which was superior to the sensitivity (77.3%) and specificity (60.5%) obtained by the original biomarker panel in our previous study [13].

As an AUC of 0.5 indicates a valueless test and an AUC of 1.0 indicates an excellent test, these results demonstrate that the two urinary metabolite panels are “good” classifiers of male BD and female BD subjects.

### Sex-Specific Biomarker Assessment

In order to assess the specificity of the identified potential sex-specific biomarkers, the male group was used as the test set for the female group and vice versa. The results showed that the panel composed of the biomarkers identified in the male group had a relatively low discrimination power in the female group with a 77.3% sensitivity and a 58.1% specificity, and the panel composed of the biomarkers identified in the female group also had a relatively low discrimination power in the male group with a 66.7% sensitivity and a 79.2% specificity.

## Discussion

Currently, no objective lab-based diagnostic test is available to aid clinicians in making an accurate diagnosis of BD. Although our previous study had identified a potential urinary metabolite biomarker panel for diagnosing BD, this panel could not effectively discriminate on a sex-based basis. Here, a urinary metabonomic profiling study was carried out in search of sex-specific metabolite biomarker panels for BD. Two urinary metabolite biomarker panels separately consisting of four metabolites (N-methylnicotinamide, formate, α-hydroxybutyrate, and choline) for males and four metabolites (N-methylnicotinamide, acetone, α-hydroxybutyrate, and oxalacetate) for females were identified “good” classifiers with AUCs of 0.942 and 0.909, respectively. The male-specific panel yielded good separation specific to male BD and HC subjects, and the female-specific panel yielded good separation specific to female BD and female HC subjects. Meanwhile, several other metabolites had different trends between male and female BD subjects (Table 2). These findings suggest that male and female BD patients have distinct biomarker fingerprints, which can be detected in urine. These two panels may facilitate the development of a urine-based diagnostic test for diagnosing male and female BD patients and may provide a window into the mechanisms underlying the pathoetiology of BD in both men and women.

In male BD subjects, we observed increased levels of α-hydroxybutyrate and formate as well as decreased levels of N-methylnicotinamide and choline. In female BD subjects, we observed increased levels of α-hydroxybutyrate as well as decreased levels of N-methylnicotinamide, acetone, and oxalacetate. Although the levels of α-hydroxybutyrate and formate were not significantly changed in the univariate statistical analysis, they were still included in the diagnostic biomarker panel because the stepwise regression analysis showed that, by adding these two metabolites, the composite panel would result in the highest predictive power. These results demonstrate the advantage of a multivariate statistical approach in detecting subtle yet significant metabolic differences between experimental groups above and beyond a simple univariate analysis [20].

Although choline and isobutyrate were identified as potential biomarkers in our previous study, these two metabolites were not identified here as potential metabolite biomarkers in discriminating female BD and HC subjects [13]. Isobutyrate was significantly increased in BD subjects in the previous study, but in this study, we found that isobutyrate was significantly increased in male BD subjects and non-statistically significantly decreased in female BD subjects. Moreover, acetone, which was identified as a potential female-specific biomarker in this study, was significantly increased in female BD subjects but non-statistically significantly increased in male BD subjects. Moreover, several other metabolites displayed different trends between male and female BD subjects (Table 2). These results indicate that it is prudent to separately identify potential urinary metabolite biomarkers for diagnosing BD on a sex-specific basis.

Considering the low discriminatory power of our previously identified biomarkers in females, the current work shows promise in aiding the effective diagnosis and treatment of female BD patients. BD is relatively rare in obstetrics and gynecology practice as compared to depressive and anxiety disorders, but there continues to be a high risk of poor outcomes for female BD patients and their offspring [21]. Teatero et al. reported that female BD patients have increased rates of premenstrual dysphoric disorder [22]. With respect to pregnant women, little is known about the course and treatment of BD during pregnancy and the postpartum period [23], and the postpartum period is generally considered to be a period of heightened vulnerability to BD [24]. Therefore, an objective and accurate diagnostic tool for female BD subjects is urgently needed. Our previous work identified a female-specific biomarker panel with an accuracy of 68.9% in discriminating female BD and HC subjects [13], but this work yielded a female-specific biomarker panel with a superior accuracy of 82.8%. This new biomarker panel can contribute to the development of a more personalized diagnosis and treatment experience for female BD patients.

To analyze the biological functions of these differential metabolites responsible for distinguishing BD subjects from HC, the pathway analysis was performed by online software MetaboAnalyst. For male, the differential metabolites were mainly involved in four metabolic pathways: synthesis and degradation of ketone bodies, methane metabolism, phenylalanine metabolism, and tyrosine metabolism. For female, the differential metabolites were mainly involved in four metabolic pathways: synthesis and degradation of ketone bodies, methane metabolism, glycerophospholipid metabolism and glycine, serine and threonine metabolism. The relationship of these pathways with affective disorder was also reported by previous studies. One study reported that synthesis and degradation of ketone bodies was the most influenced metabolic pathways associated with CUMS-induced depression [25]. The level of formate and dimethylamine in methane metabolism was found to be significantly changed in BD subjects [13]. Phenylalanine and tyrosine depletion could selectively lower dopamine (DA) synthesis, which was involved in unipolar depression [26]. Farooqui et al reported that marked alterations in neural membrane glycerophospholipid composition occurred in neurological disorders [27]. The serum levels of glycine, serine and threonine were significantly changed in treatment-resistant depression [28].

The results of this study should be cautiously interpreted on account of the following limitations. First, the relevance of the identified biomarkers to the pathogenesis of BD should be investigated by collecting and analyzing cerebrospinal fluid (CSF) and post-mortem brain samples from male and female BD patients. Second, due to the limited number of included BD patients and clinical sites in the current study, further studies consisting of larger sample sizes across multiple centers are required to verify and support the diagnostic efficacy of these biomarkers. Third, the small sample size did not allow for subgroup comparisons. Further studies are needed to determine whether the two sex-specific panels can be used to distinguish the three MD mood states. Fourth, this study did not compare BD subjects against other neuropsychiatric disorders of similar clinical presentation such as depression; further studies should focus on whether these biomarkers can be applied to differentiate BD from other such neuropsychiatric disorders. Fifth, this work did not assess the influences of psychotropic drugs on the identified biomarkers. Finally, due to the diverse physicochemical properties and wide concentration range of metabolite biomarkers, future studies should employ additional metabolomic methods or multiple metabolomic platforms [29], [30].

## Conclusions

In conclusion, using a NMR-based metabonomic approach, two sex-specific urinary metabolite biomarker panels for diagnosing male and female BD patients were identified here. The male-specific panel (consisting of α-hydroxybutyrate, choline, formate, and N-methylnicotinamide) and the female-specific panel (consisting of α-hydroxybutyrate, acetone, oxalacetate, and N-methylnicotinamide) were both capable of differentiating male and female BD subjects from their respective HC with higher accuracy than our previously identified biomarker panel. These findings suggest that male and female BD patients have distinct biomarker fingerprints and that these two sex-specific biomarker panels may serve as effective diagnostic tools in distinguishing male and female BD patients from their healthy counterparts.

## Supporting Information

S1 File **Original data about the age, body mass index and metabolites.**
(XLSX)
